# Modulating Chikungunya and Mayaro virus-induced disease severity in mice using low concentrations of anti-IFNAR1 antibodies

**DOI:** 10.1080/22221751.2025.2611479

**Published:** 2026-01-14

**Authors:** Konrad Wesselmann, Léa Luciani, Gregory Moureau, Jean-Selim Driouich, Ornellie Bernadin, Magali Gilles, Xavier de Lamballerie, Antoine Nougairède

**Affiliations:** a Unité des Virus Émergents (UVE: Aix-Marseille Univ, Università di Corsica, IRD 190, Inserm 1207, IRBA), Marseille, France; b Assistance Publique-Hôpitaux de Marseille (AP-HM), Service de Virologie Aiguë et Tropicale, Marseille, France; c National Reference Center for Arboviruses, Inserm-IRBA, Marseille, France

**Keywords:** Chikungunya virus, Mayaro virus, mice, anti-IFNAR1, immunocompromised

## Abstract

The laboratory mouse (*Mus musculus*) is the most widely used animal model for preclinical research, with numerous wild-type and genetically modified mouse strains available. Chikungunya virus (CHIKV), a mosquito-borne arthritogenic alphavirus, has emerged in various new regions and caused several millions of cases within the last decade. Mayaro virus (MAYV), an arthritogenic alphaviruses closely related to CHIKV, remains geographically restricted to the Americas. Existing mouse models rely on immunodeficient mice, leading to lethal illness, or footpad injection, which induces localized arthropathy. We present a proof-of-concept study demonstrating how disease severity in mice can be modulated using sub-neutralizing concentrations of an interferon 1 receptor (IFNAR1) blocking monoclonal antibody (mAb). C57BL/6 mice were injected intraperitoneally with varying anti-IFNAR1 antibody doses before intraperitoneal infection with CHIKV or MAYV. For both, CHIKV and MAYV, we observed an anti-IFNAR1 mAb dose-dependent increase in blood viral loads and disease severity. A 1mg dose induced severe disease, whereas a 0.1 mg dose resulted in moderate symptoms in mice, mainly facial pain expression signs, accompanied by detectable viremia in the days preceding symptom onset. Viral loads in organs and serum concentrations of inflammatory cytokines and chemokines were also elevated in mice receiving 0.1mg anti-IFNAR1 mAb. In conclusion, we provide proof of concept that CHIKV and MAYV disease severity can be modulated using low concentrations of anti-IFNAR1 mAb. We used this approach to develop a new infection model for mild systemic disease, based on an accessible strain and a commercial antibody allowing for easy implementation and adaptation.

## Introduction

A previous version of this manuscript has been published as a preprint: https://doi.org/10.21203/rs.3.rs-6812821/v1 [1].

Alphaviruses are positive-sense single-stranded enveloped RNA viruses of the family *Togaviridae*. Depending on their pathogenicity in humans, they are classified into two groups: encephalitic and arthritogenic [2]. Chikungunya virus (CHIKV), Mayaro virus (MAYV), and o’nyong-nyong virus (ONNV) are antigenically related arthritogenic alphaviruses belonging to the Semliki Forest complex [3]. CHIKV is of particular concern because of its recurrent re-emergence and its recent geographic expansion. Originally causing few outbreaks in Africa and Asia, CHIKV re-emerged in 2005, causing major outbreaks in the Indian Ocean, then in Southeast Asia [4]. Between 2013 and 2015, several CHIKV lineages were introduced to the Americas, causing large-scale epidemics [5]. The adaptation of the East-Central-South African genotype (ECSA) to the *Aedes albopictus* mosquito vector enabled further spread of the virus into more temperate climate zones, notably in Europe [4]. Currently, millions of people live in endemic areas [6]. Unlike most other arboviral diseases, CHIKV infection is symptomatic in most cases (between 70 and 80%), and almost half of these people go on to develop persistent, debilitating forms of arthralgia [7]. There is currently no specific anti-viral treatment for this debilitating disease. Though the first vaccine against CHIKV received FDA approval in 2023. MAYV and ONNV also cause potentially persistent arthralgia, but their circulation is currently restricted by the geographical distribution of their respective vectors: South America for MAYV [8] and sub-Saharan Africa for ONNV [9]. Thus, in these regions, there is co-circulation with CHIKV.

Experimental animal models are crucial for studying these viruses, particularly for analysing their pathogenesis and evaluating the efficacy of new antiviral therapies. Murine models of arthritogenic alphaviruses are difficult to set up because wild-type mice may not develop clinical signs. Several murine models for acute arthritogenic alphavirus infections have been developed. They can be attributed to three categories: arthritis/myositis model, neonatal models, and knockout mouse models [10]. Infection of mice using the arthritis/myositis model occurs by intradermal or subcutaneous injection of the virus into the footpad of one of the hindlegs. Wilde type (WT) mice infected with CHIKV using this model show a biphasic footpad swelling in the injected foot and measurable viremia. The arthritis/myositis model is the well-established reference model for arthralgia induced by alphaviruses [10,11]. In contrast, infection of newborn mice with CHIKV is uniformly fatal [10]. It is generally accepted that the innate immune response, especially the interferon response, is critical for early control of virus dissemination of arthritogenic alphaviruses. Commonly used knockout (KO) mouse models include type I interferon (IFN-I)receptor-deficient mice (A129) and mice lacking both IFN-I and IFN-II receptors (AG129).

ONNV infection results in approximately 50% lethality in mice with KO-mutations of the Interferon α/β receptor (IFNAR) (A129 mice)[12], whereas CHIKV and MAYV infections lead to 100% mortality [10,13]. KO mouse strains, however, are not widely available and permanently immunodeficient mice can be incompatible with some experimental designs. As an alternative approach, the blocking of mice IFN-I receptor (IFNAR) using the monoclonal antibody (mAb) MAR1-5A3 targeting the IFNAR subunit one (IFNAR1) of the receptor has been explored for a couple of other arboviruses, such as Zika, dengue, Crimean-Congo hemorrhagic fever viruses, as well as CHIKV [14–17]. The antibody has a short serum half-life of 5 days when administered at saturating doses. This duration is reduced to 1.5 days when given at sub-saturating doses, presumably due to substantial intracellular reservoirs of IFNAR1 cycling to the surface [18]. In this study, we provide proof of concept that the disease severity of alphavirus infections in mouse models can be modulated by injecting varying doses of anti-IFNAR1 mAb. We use CHIKV and MAYV to propose a straightforward experimental approach which could be adopted for other viruses. Finally, we characterize a new model for mild systemic CHIKV or MAYV infection, complementing existing infection models.

## Material and methods

### Cells

Cells were prepared as previously described [19]. Vero E6 cells (ATCC CRL-1586, RRID: CVCL_0574) were obtained from ATCC. Upon receipt, cells were thawed, amplified, and stored in working aliquots according to the supplier’s recommendations. Cells tested negative for mycoplasma contamination. Cells were cultured at 37°C with 5% CO_2_ in minimal essential medium (MEM; Life Technologies) supplemented with 7% heat-inactivated fetal bovine serum (FBS; Life Technologies), 1% penicillin/streptomycin (PS; 5000U/mL and 5000μg/mL; Life Technologies), and 1% glutamine (Gln; 200 mmol/L; Life Technologies).

### Viruses

Virus stocks were prepared as previously described [19]. CHIKV LR2006_OPY1 (CHIKV OPY1), MAYV UVE/MAYV/1954/TT/TC625, and ONNV UVE/ONNV/UNK/SN/Dakar-234 were kindly provided by the European Virus Archive GLOBAL (EVAg; https://www.european-virusarchive.com/). Virus working stocks were generated by inoculation of confluent Vero E6 cells in 75cm^2^ culture flasks at a multiplicity of infection (MOI) of 0.001 (CHIKV, ONNV) and 0.01 (MAYV) in MEM supplemented with 2.5% FBS, 1% PS and 1% Gln. After 1 h of inoculation time, cells were washed and the media replaced. Viruses were harvested upon the first appearance of cytopathic effects, filtered (45 µm), supplemented with 25 mM HEPES (Sigma-Aldrich), aliquoted and stored at −80°C. Infectious titres were determined from two independent aliquots. Tenfold serial dilutions were prepared, and confluent VeroE6 cells on 96-well plates were inoculated. Each dilution was tested in sextuplicate. Plates were incubated for 7 days, after which cytopathic effects were determined and 50% tissue-culture infectious dose (TCID_50_) titres were calculated according to the method of Reed and Muench [20]. All experiments with infectious virus were conducted in a biosafety level 3 (BSL3) facility.

### In vivo experiments

*In vivo* experiments were approved by the local ethical committee (C2EA–14) and the French “Ministère de l’Enseignement Supérieur, de la Recherche et de l’Innovation” (APAFIS #39524) and performed in accordance with the French national guidelines and the European legislation covering the use of animals for scientific purposes. This study was conducted according to ARRIVE guidelines (https://arriveguidelines.org/).

*Animal handling*. Six-week-old female C57BL/6 mice (strain code 027) were provided by Charles-River Laboratories. Animals were maintained in ISOcage P – Bioexclusion System (Techniplast) with unlimited access to water/food and a 14 h/10 h light/dark cycle. A wooden gnawing block and extra bedding material were provided as cage enrichment. Experiments started after one week of acclimatization. Animals were weighed and monitored daily for the duration of the study to detect the appearance of any clinical signs of illness and suffering. A cumulative score with points for specific symptoms, taking into account the respective symptoms' severity, was used to assess disease severity for each animal. Points were given for pilo-erection (1 point), hunched posture (1 point), orbital tightening (1 point), ear positions facing outwards away from the indicating pain (1 point), hypo-reactivity (2 points), lethargy (5 points), pale mucous membranes (5 points), moribund, generalized trembling, or loss of movement control (10 points). We noticed that the weight of the mice was quite variable, and small weight losses did not correlate with clinical disease or viral loads. Thus, only significant weight losses (weight losses >15% or >20%) were considered as signs of clinical disease (5 and 10 points, respectively). Weight curves of individual mice are displayed in Figures S1–S7. Mice with a cumulative score of 10 or more were euthanized. Injections, blood sampling and euthanasia by cervical dislocation were performed under general anaesthesia obtained with isoflurane (Isoflurin®, Axience).

**Figure 1.**
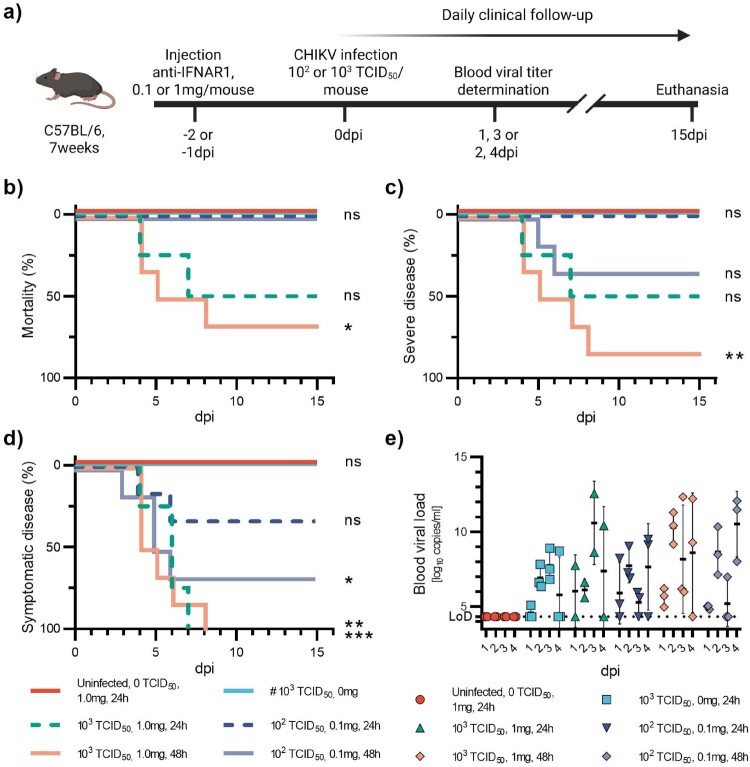
Pilot study on CHIKV. 7-week-old C57BL/6J mice were injected with high (1 mg) or low (0.1 mg) doses of anti-IFNAR1 mAb 24 h or 48 h before infection with 10^3^TCID_50_ or 10^2^TCID_50_ of CHIKV (a). Two control groups received 1 mg anti-INAR1 mAb and a mock infection or 0 mg anti-IFNAR1 mAb and 10^3^TCID_50_ of CHIKV. Mortality (based on humane endpoints) (b), appearance of severe disease (c) and symptomatic disease (d) were monitored daily after infection for 15 days. Viral loads were measured at days post-infection one and three, or two and four in half the mice, respectively (e). Corresponding serology results are displayed in Figure S8a and Table S1a. Data are represented as Kaplan-Meier curves (b-d) or mean ± SD (e). Two-sided statistical analysis was performed using the Log-rank (Mantel-Cox) test (b-d) (details in Table S2). *, ** and *** mean *p*-value ranging between 0.01−0.05, 0.05–0.001 and 0.001–0.0001, respectively. ****: *p*-value ≤0.0001. ns: not significant. If not otherwise indicated, comparisons were made with the #-marked group.

**Figure 2.**
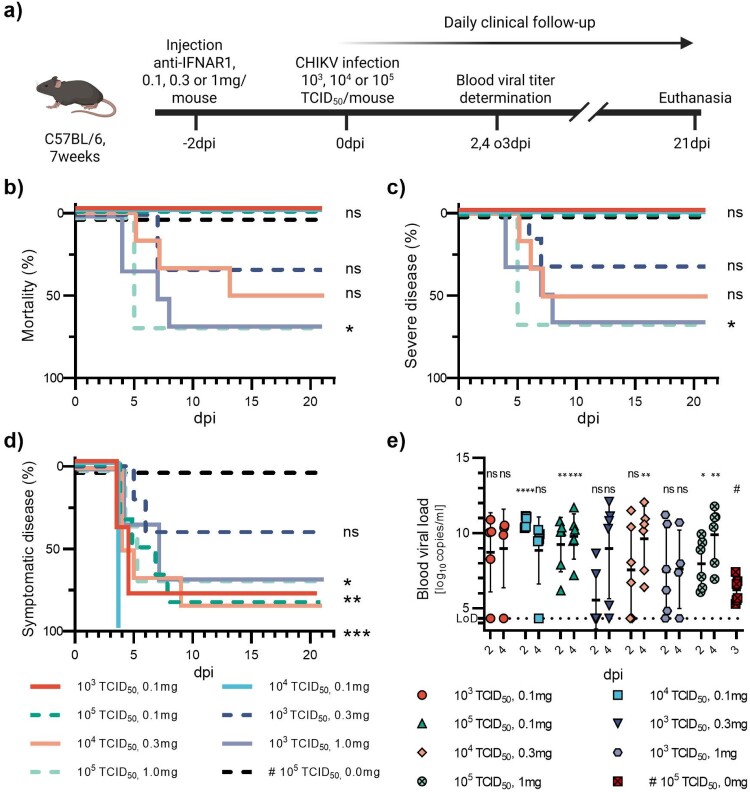
CHIKV infection with varying infectious doses and varying preceding doses of anti-IFNAR1 mAb. 7-week-old C57BL/6J mice were injected with anti-IFNAR1 mAb (doses varying from 0.1 to 1 mg) 48 h before infection with varying CHIKV doses (10^3^TCID_50_ or 10^5^TCID_50_) (a). A control group received 0 mg anti-IFNAR1 mAb and 10^5^TCID_50_ CHIKV. Mortality (based on humane endpoints) (b), appearance of severe disease (c) and symptomatic disease (d) were monitored daily after infection for 21 days. Viral loads were measured at days post-infection two and four (e). Corresponding serology results are displayed in Figure S8b and Table S1b. Data are represented as Kaplan-Meier curves (b-d) or mean ± SD (d). Two-sided statistical analysis was performed using Log-rank (Mantel-Cox) test (b-d) or Shapiro–Wilk normality test followed by Student t-test, Student t-test with Welch correction or Mann-Whitney test (e) (details in Table S3). *, ** and *** mean *p*-value ranging between 0.01−0.05, 0.05–0.001 and 0.001–0.0001, respectively. ****: *p*-value ≤0.0001. ns: not significant. If not otherwise indicated, comparisons were made with the #-marked group.

**Figure 3.**
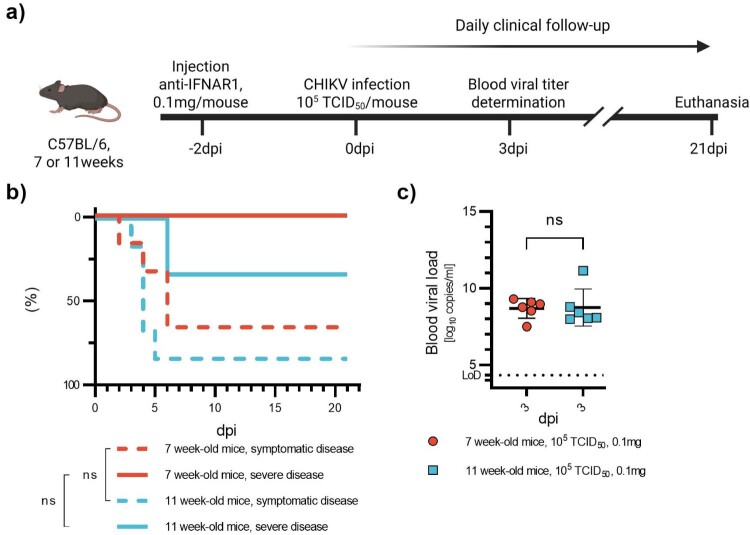
CHIKV infection in young and mature adult mice. 7- or 11-week-old C57BL/6J mice were injected with anti-IFNAR1 mAb (0.1 mg) 48 h before infection with CHIKV (10^5^TCID_50_) (a). Mortality (based on humane endpoints, not shown due to the absence of events), appearance of severe disease and symptomatic disease (b) were monitored daily after infection for 21 days. Viral loads were measured at day post-infection three (c). Corresponding serology results are displayed in Figure S8c and Table S1c. Data are represented as Kaplan-Meier curves (b) or mean ± SD (c). Two-sided statistical analysis was performed using Log-rank (Mantel-Cox) test (b) or Shapiro–Wilk normality test followed by Mann-Whitney test (c) (details in Table S4). *, ** and *** mean *p*-value ranging between 0.01−0.05, 0.05–0.001 and 0.001–0.0001, respectively. ****: *p*-value ≤0.0001. ns: not significant.

**Figure 4.**
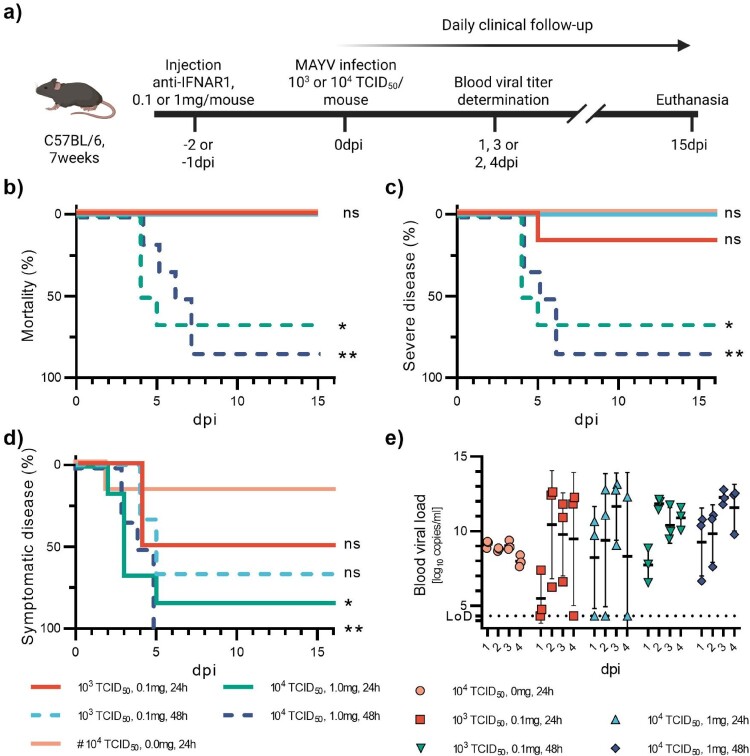
Pilot study on MAYV. 7-week-old C57BL/6J mice were injected with very high (1 mg) or very low (0.1 mg) doses of anti-IFNAR1 mAb 24 h or 48 h before infection with 10^3^TCID_50_ or 10^4^TCID_50_ of CHIKV (a). A control group received 0 mg anti-IFNAR1 mAb and 10^4^TCID_50_ of MAYV. Mortality (based on humane endpoints) (b), appearance of severe disease (c) and symptomatic disease (d) were monitored daily after infection for 17 days. Viral loads were measured at days post-infection one and three, or two and four in half the mice, respectively (e). Corresponding serology results are displayed in Figure S8d and Table S1d. Data are represented as Kaplan-Meier curves (b-d) or mean ± SD (e). Two-sided statistical analysis was performed using the Log-rank (Mantel-Cox) test (b-d) (details in Table S5). *, **, and *** mean *p*-value ranging between 0.01−0.05, 0.05–0.001, and 0.001–0.0001, respectively. ****: *p*-value ≤0.0001. ns: not significant. If not otherwise indicated, comparisons were made with the #-marked group.

**Figure 5.**
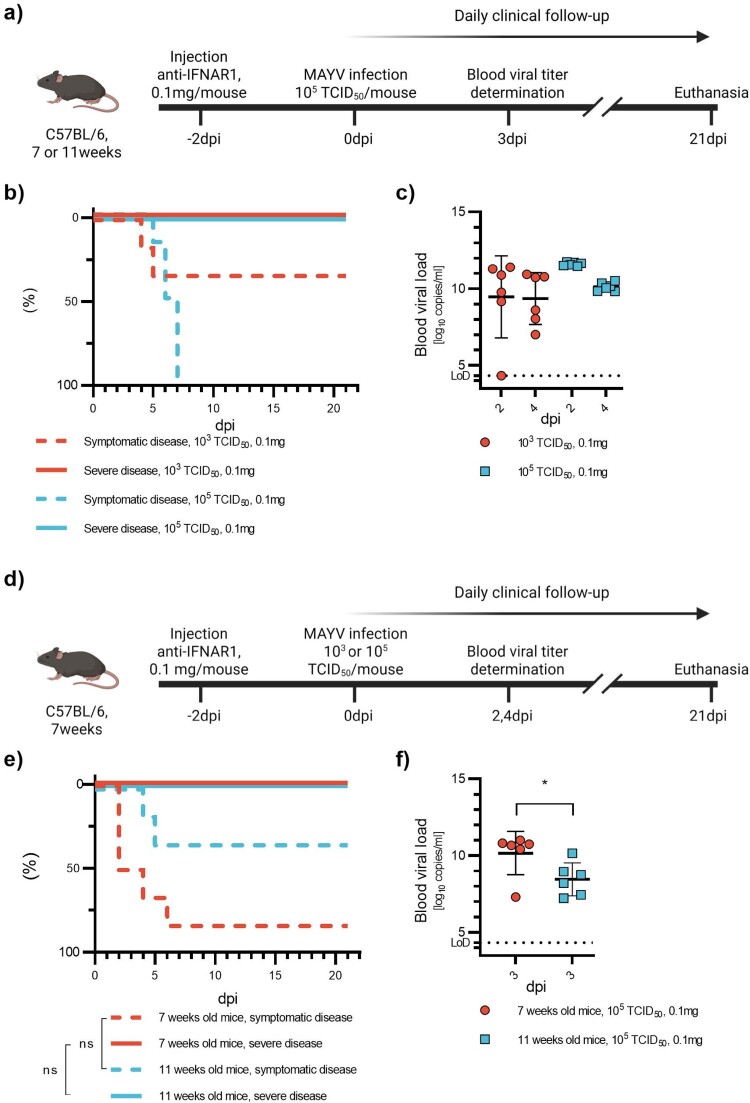
Establishment of the MAYV infection model. Infection with 10^3^TCID_50_ or 10^5^TCID_50_ and a preceding dose of 0.1 mg anti-IFNAR1 mAb (a–c) and MAYV infection in young and mature adult mice with a preceding dose of 0.1 mg anti-IFNAR1 mAb and a high viral titre of 10^5^TCID_50_ (d–f). 7-week-old C57BL/6J mice were injected with anti-IFNAR1 mAb (0.1 mg) 48 h before infection with varying MAYV doses (10^3^TCID_50_ or 10^5^TCID_50_) (a). Mortality (based on humane endpoints, not shown due to absence of events), appearance of severe disease and symptomatic disease (b) were monitored daily after infection for 21 days. Viral loads were measured at days post-infection two and four (c). Corresponding serology results are displayed in Table S1e. 7- or 11-week-old C57BL/6J mice were injected with anti-IFNAR1 mAb (0.1 mg) 48 h before infection CHIKV (10^5^TCID_50_) (d). Mortality (based on humane endpoints, not shown due to the absence of events), appearance of severe disease and symptomatic disease (e) were monitored daily after infection for 21 days. Viral loads were measured at day post-infection three (f). Corresponding serology results are displayed in Figure S8f and Table S1f. Data are represented as Kaplan-Meier curves (b and e) or mean ± SD (c and f). Two-sided statistical analysis was performed using the Log-rank (Mantel-Cox) test (b and e) or the Shapiro–Wilk normality test followed by the Mann-Whitney test (f) (details in Table S6). *, ** and *** mean *p*-value ranging between 0.01−0.05, 0.05–0.001 and 0.001–0.0001, respectively. ****: *p*-value ≤0.0001. ns: not significant.

**Figure 6.**
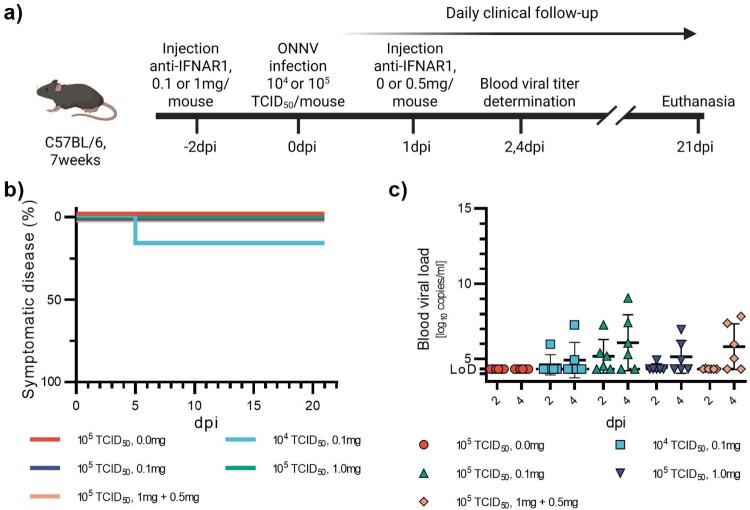
Low susceptibility of mice to ONNV infection. 7-week-old C57BL/6J mice were injected with anti-IFNAR1 mAb (doses varying from 0.1 to 1 mg) 48 h before infection with ONNV (10^4^TCID_50_ or 10^5^TCID_50_) (a). One group received 1 mg anti-IFNAR1 mAb 48 h pre-infection with 10^5^TCID_50_ and 0.5 mg 24 h post-infection. Mortality (based on humane endpoints), appearance of severe disease and symptomatic disease were monitored daily after infection for 21 days (b). Viral loads were measured at days post-infection two and four (c). Corresponding serology results are displayed in Figure S8g and Table S1g. Data are represented as Kaplan–Meier curves (b) or mean ± SD (c).

**Figure 7.**
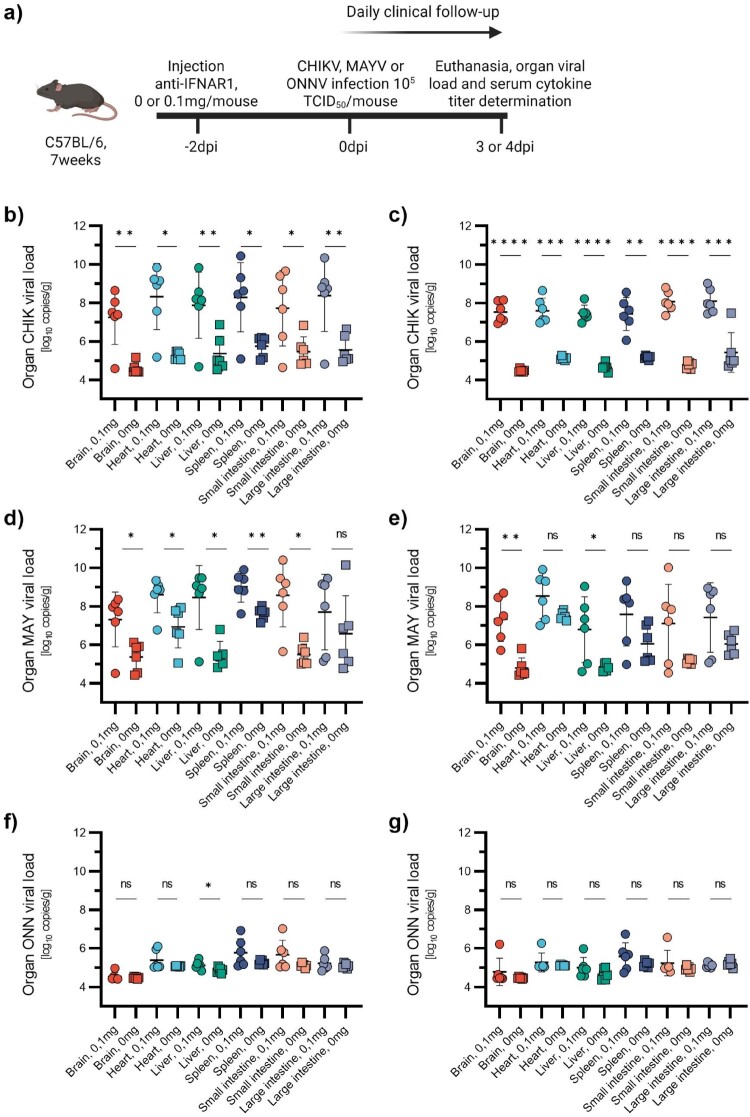
Viral loads in various organs after infection with the established model. 7-week-old C57BL/6J mice were injected with anti-IFNAR1 mAb (0.1 mg) 48 h before infection with CHIKV, MAYV, or ONNV (10^5^TCID_50_) (a). Mice were euthanized at 3 (b, d and f) or 4 (c, e and g) dpi and viral loads in brain, heart, liver, spleen, small intestine, and large intestine were determined by RT-qPCR. Data are represented as mean ± SD. Two-sided statistical analysis was performed using the Shapiro–Wilk normality test, followed by Student t-test, Student t-test with Welch correction or Mann-Whitney test (details in Table S7). *, ** and *** mean *p*-value ranging between 0.01 and 0.05, 0.05–0.001 and 0.001–0.0001, respectively. ****: *p*-value ≤0.0001. ns: not significant.

**Figure 8.**
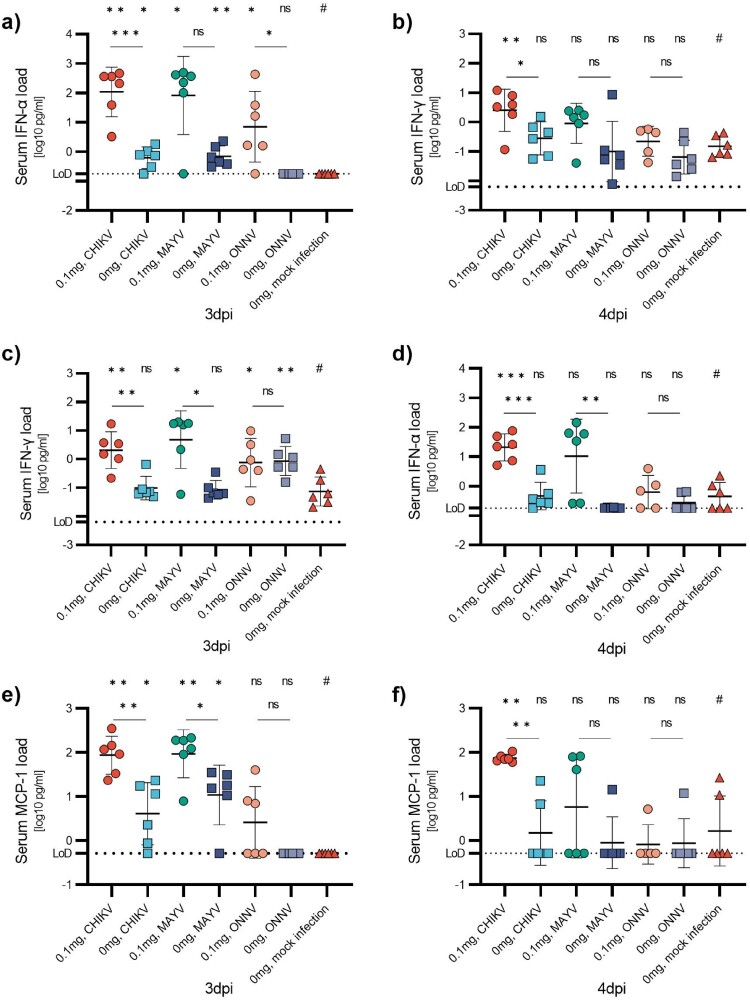
Serum cytokine/chemokine concentrations after infection with the established model. 7-week-old C57BL/6J mice were injected with anti-IFNAR1 mAb (0.1 mg) 48 h before infection with CHIKV, MAYV or ONNV (10^5^TCID_50_). Mice were euthanized at 3 (a, c, and e) or 4 (b, d, and f) dpi and serum cytokine concentrations were determined using ProQuantum high-sensitivity immunoassays. Data are represented as mean ± SD. Two-sided statistical analysis was performed using the Shapiro–Wilk normality test followed by Student *t*-test, Student *t*-test with Welch correction or Mann-Whitney test (details in Table S9). *, ** and *** mean *p*-value ranging between 0.01−0.05, 0.05–0.001, and 0.001–0.0001, respectively. ****: *p*-value ≤0.0001. ns: not significant.

*Study design*. We employed a basic infection model: intraperitoneal (ip) injection, clinical/weight monitoring and tail blood sampling. To find an animal model with systemic yet moderate disease, as can be observed in the majority of human alphavirus infections, we varied three parameters: the dose of virus injected (from 10^2^ to 10^5^ TCID_50_), the dose of anti-IFNAR injected (from 0.1 mg to 1 mg initial plus 0.5 mg follow-up) and the time between IFNAR injection and viral infection (24 or 48 h). Group size was calculated with an effect size of 2 and a power of 80%, resulting in 5–6 animals/group. Groups of 6 animals per group were used in all experiments, except for the groups 10^3^TCID_50_, 1 mg, 48 h in the pilot study on CHIKV (Figure 1) and the group 10^3^TCID_50_, 0.1 mg in the experiment investigating CHIKV infection with varying infectious doses and varying preceding doses of anti-IFNAR1 mAb (Figure 2) which had grouped sizes of 4 and 5 respectively. Animals of the same group were held together in a single cage. A total of 261 animals were used in this study: 34 animals were used for a pilot study on CHIKV (Figure 1); 47 animals were used for investigating CHIKV infection with varying infectious doses and varying preceding doses of anti-IFNAR1 mAb (Figure 2); 12 animals were used investigating CHIKV infection in young and mature adult mice (Figure 3); 30 animals were used for a pilot study on MAYV (Figure 4); 24 animals were used for the establishment of a MAYV infection model and for investigating MAYV infection in young and mature adult mice (Figure 5); 30 animals were used for investigating the susceptibility of mice for ONNV infection (Figure 6); and 84 animals were used for investigating viral loads in various organs and serum cytokine/chemokine concentrations after infection with the established model (Figures 7 and 8). Animals were randomly assigned to groups, but confounders were not controlled. Since the same experimenters carried out infection/treatment/clinical follow-up, it was impossible to perform a blind trial. Inexplicable death was set as an exclusion criterion. No animals were excluded from the study.

*Anti-IFNAR1 mAb injection*. Mouse anti-IFNAR1 mAb (MAR1-5A3) was purchased from Leinco Technologies Inc. Upon receipt, the antibody was aliquoted and stored at −80°C. The antibody was administered ip (100 µl diluted in 0.9% sodium chloride solution). Mice that received 0 mg of anti-IFNAR mAb were injected with saline solution only.

*Infection*. Seven weeks-old anesthetized animals were intraperitoneally infected with 100 µL containing different doses of virus in 0.9% sodium chloride solution. Mock-infected groups were inoculated with 100 µL of 0.9% sodium chloride solution.

*Blood sampling on live infected mice*. Anaesthetized animals were bled by caudal artery puncture. Approximately 10 µl of blood was diluted in 90 µl of 0.9% sodium chloride solution containing 0.01M EDTA. When we monitored viremia kinetics daily for the first 4 days post-infection (dpi), for animal welfare reasons, we limited the number of blood collections per mouse. For each group, half of the mice were sampled at 1 and 3 dpi, while the other half were sampled at 2 and 4 dpi. This approach allowed for daily monitoring of viremia for each group while ensuring a 48-hour interval between blood collections for each animal.

### Quantitative real-time RT–PCR (RT-qPCR) assays

RT-qPCRs were conducted as previously described [19]. All experiments were conducted in a molecular biology facility dedicated to molecular clinical diagnostics, with separate laboratories assigned to each procedural step. Prior to PCR amplification, RNA was extracted using the QIAamp 96 DNA kit and the Qiacube HT kit and the Qiacube HT system (Qiagen) according to the manufacturer's instructions. Briefly, 100 µl of tissue clarified homogenates (prepared as previously described [17]), spiked with 10 µl of internal control (bacteriophage MS2), or 50 µl of 10-fold diluted blood, were transferred into an S-block containing the recommended volumes of VXL buffer, proteinase K and RNA carrier. RT-qPCR assays for CHIKV, MAYV, ONNV and MS2 internal control were performed using SuperScript® III Platinum® One-Step RT-qPCR Kit with ROX (#11732-088, Invitrogen-Thermo Fisher Scientific, Waltham, MA, USA). Primer and probe sequences and concentrations for the detection of CHIKV [21], MAYV [22] and ONNV [23] were applied as reported in the literature. Quantification was achieved using four tenfold serial dilutions of known concentrations (10^7^ to 10^4^ copies/reaction) of T7-generated synthetic RNA standards. Amplification was performed on the QuantStudio 12 K Flex Real-Time PCR System (Applied Biosystems) with the following cycling conditions: 15 min at 50°C, 2 min at 95°C, and 40 cycles of 95°C for 15 sec followed by 45 s at 60°C. Data were analysed using QuantStudio 12 K Flex software v1.2.3 (Applied Biosystems). Positive controls, primers and probes were kindly provided by the European Virus Archive-Marseille (EVAM) under the label technological platforms of Aix-Marseille (https://evam.european-virus-archive.com/).

### ELISA assay

Measurement of serum levels of specific IgG was performed for CHIKV and ONNV using the kit “Anti-Chikungunya Virus ELISA (IgG)” (EUROIMMUN Medizinische Labordiagnostika AG, Lübeck, Germany) according to the manufacturer’s instructions. Measurement of serum levels of specific IgG was performed for MAYV using the kit “Anti-Mayaro Virus ELISA (IgG)” (EUROIMMUN Medizinische Labordiagnostika AG, Lübeck, Germany) according to the manufacturer’s instructions. With both kits, and in order to detect mouse IgG, the secondary antibody was replaced by Goat anti-Mouse IgG (H + L) secondary Antibody, HRP (#32430, Invitrogen-Thermo Fisher Scientific, Waltham, MA, USA), diluted 1:500 in HBSS, 1% BSA. Optical density (OD) at 450 nm was measured. Samples with an OD_450_ > 0.1 were considered positive. ELISA results are displayed in Figure S8 and Table S1.

### Quantification of serum cytokine levels

Serum cytokine levels were measured using Proquantum immunoassays (#A41150, #A43656, #A43658, #A44837 and #A46736, Invitrogen-Thermo Fisher Scientific, Waltham, MA, USA) according to the manufacturer’s instructions. The PCR step of the assay was performed on a BioRad CFX96TM thermal cycler, software version 3.1 (Bio-Rad Laboratories, Hercules, CA, USA).

### Graphical representations and statistical analysis

Graphical representations and statistical analyses were performed similarly to previous work [19] using Graphpad Prism version 9.4.1 (Graphpad software). Statistical comparisons were conducted using Log-Rank and Mann–Whitney tests with *p*-values < 0.05 considered statistically significant. Detailed information on the performed statistics for each experiment is provided in the figure legends. Experimental timelines were created in https://BioRender.com.

## Results

### CHIKV infection severity in mice can be modulated using varying doses of anti-IFNAR1 mAb doses

In a first series of experiments, we explored the effect of various anti-IFNAR1 doses on CHIKV disease severity in mice. We first investigated differences between injection 24 h and 48 h and tested two conditions, which we suspect to induce severe or mild to no disease. Based on the results of the first experiment, we investigate different doses of antibodies and virus in a second step. For this, 7-week-old female C57BL/6 mice are first treated with anti-IFNAR1 mAb (ip, varying doses, 24 h or 48 h pre-infection) and then infected with CHIKV (ip, varying doses, CHIKV ECSA genotype). Blood viral loads were measured in the days following infection by collecting blood samples. Mice were monitored daily for 15 days in the initial experiment and later for 21 days for signs of viral disease. The monitoring period was extended after the initial experiments due to persisting symptoms. On the basis of a clinical assessment grid detailed in the materials and methods section, survival curves for (i) mortality (based on humane endpoints), (ii) onset of severe disease (score >4 at least one day), and (iii) symptomatic disease (moderate or severe) (score >1 at least on day or =1 two consecutive days) were generated.

In the initial experiment, we aimed to define the two extreme conditions of our model (severe disease and mild to no disease) based on the literature [24,25] and the manufacturer’s guidelines for the anti-IFNAR1 mAb [26]. The extreme doses of anti-IFNAR1 mAb were set at 0.1 and 1 mg per mouse and associated with viral doses of 10² or 10³ TCID₅₀, respectively. Both conditions were evaluated with a 24 h or 48 h interval between injections (Figure 1(a)). We also had two control groups, one of uninfected animals receiving the highest dose of anti-IFNAR alone (1 mg) and a second of animals infected with 10^3^ TCID_50_ but receiving no anti-IFNAR mAb. Overall, infection in mice receiving 1 mg of anti-IFNAR1 mAb 48 h before the infection was more severe (66% mortality, 83% severe disease) compared to those receiving the same dose of mAb 24 h before infection (50% mortality and severe disease) (Figure 1(b,c)). Some mice receiving the lowest dose of anti-IFNAR1 mAb developed mild disease characterized mainly by facial expression of pain. The proportion of symptomatic mice was also higher when anti-IFNAR1 mAb was administered 48 h before infection (66% versus 33%) (Figure 1(d)). Symptom onset occurred between 3 and 10 dpi. None of the mice in either of the control groups exhibited any clinical signs. Peak blood viral loads were observed between 2 and 4 dpi in all groups. However, viral loads exhibited important variability across all groups and time points, with SDs spanning several orders of magnitude (Figure 1(e)). Based on the results of this pilot experiment, we decided to retain a 48-hour interval between the injection of anti-IFNAR1 mAb and infection, while also using higher viral doses for the further development of the model.

In a second experiment, we evaluated the effect of three doses of anti-IFNAR1 mAb (0.1, 0.3 and 1.0 mg per mouse) in combination with three viral doses (10^3^, 10^4^ and 10^5^ TCID_50_); not all combinations have been tested, Figure 2(a). We also had a group receiving no anti-IFNAR1 mAb and the highest viral dose (10^5^ TCID_50_). Overall, symptom onset occurred between 4 and 10 dpi. The two groups receiving the highest dose of anti-IFNAR1 mAb presented the highest rates of mortality (66% in both conditions) (Figure 2(b)). Mice receiving a viral dose of 10^3^ and 10^4^ TCID_50_ and 0.3 mg of anti-IFNAR1 mAb presented lower rates of mortality, 33% and 50%, respectively, with an additional 16% and 33% suffering from mild disease. No death or severe disease could be observed in mice receiving the lowest dose of anti-IFNAR1 (0.1 mg). However, mild disease occurred in 83%, 100%, and 83% with viral doses of 10^3^, 10^4^ and 10^5^ TCID_50,_ respectively (Figure 2(d)). None of the mice receiving no anti-IFNAR1 mAb and the highest viral dose exhibited any clinical signs. Positive viral loads were observed in most infected animals, with a tendency for the proportion of infected animals to rise as the viral dose increased. All animals that received no anti-IFNAR1 mAbs and the highest viral dose were viremic but with viral loads not exceeding 10^8^ copies/mL. Viral loads were highly variable across all groups, but this variability decreased in groups receiving higher initial viral doses (Figure 2(e)).

At this stage, it appeared that the administration of 0.1 mg per mouse of anti-IFNAR1 mAb, followed 48 h later by infection (ip) with 10^4^ or 10^5^ TCID_50_ of CHIKV represents experimental conditions inducing high viral loads (∼10^10^copies/ml) in the days following infection as well as moderate and transient illness in almost all mice (100% and 83.3%) (Figure 2(d)), and leads to seroconversion in 100% of the animals within 3 weeks (Figure 8(b) and Table S1).

In a final experiment, we assessed this condition (0.1 mg/mouse of anti-IFNAR1 monoclonal antibody administered 48 h before infection (ip) with 10^5^ TCID_50_ of CHIKV) in mature adult mice, specifically 11-week-old female C57BL/6 mice (Figure 3(a)). The aim of this experiment was to verify if anti-IFNAR doses would need to be adjusted at ages >8 weeks, since it is at that age that T-cell dependent antibody responses reach the adult levels [27]. A control group consisting of 7-week-old mice was included. Animals were bled at 3 days post-infection (dpi) and monitored daily for 21 days for signs of viral disease. No mortality or severe disease was observed in the 7-week-old group, although 66% of the animals exhibited mild symptoms, in line with results from the second experiment (Figure 3(b)). In contrast, among the 11-week-old mice, 33% developed severe disease, and an additional 50% showed mild symptoms, resulting in a total of 83% of symptomatic animals (Figure 3(b)). Similar viral loads were detected in both groups at 3 dpi (Figure 3(c)).

### MAYV infection severity in mice can be modulated using varying doses of anti-IFNAR1 mAb doses

To study the potential for modulating the severity of MAYV infection in mice after administration of anti-IFNAR1 mAbs, we used the same approach applied to CHIKV. Briefly, groups of 7-week-old female C57BL/6 mice received anti-IFNAR1 mAb (ip) one or two days before infection (ip) with doses of MAYV strain MAYV/1954/TT/TC625 (genotype D) ranging from 10^3^ to 10^5^TCID_50_. Blood viral loads were measured in the days following infection, and mice were monitored daily for 21 days. On the basis of a clinical assessment grid, survival curves were generated.

Similar to the CHIKV infection model, we aimed to define the two extreme conditions of our model (severe disease and mild to no disease) in a first experiment. Doses of 0.1 and 1 mg of anti-IFNAR1 mAb per mouse were associated with viral doses of 10^3^ or 10^4^TCID₅₀, respectively (Figure 4(a)). Both conditions were evaluated with a 24 h or 48 h interval between injections. We also had a group receiving no anti-IFNAR1 mAb and a viral dose of 10^4^TCID_50_. A pattern similar to that of CHIKV has been observed (Figure 4). Indeed, there were more diseased mice among those receiving the anti-IFNAR antibody 48 h before infection. A dose of 1 mg per mouse of anti-IFNAR1 mAb, followed by a viral dose of 10^4^TCID_50,_ elicited death in 83% and 67%, and a symptomatic disease in 100% and 83% of mice when the mAb was administered 48 h or 24 h before infection respectively. A symptomatic disease (always mild, except for one mouse) was induced in 67% and 50% of mice receiving 0.1 mg of anti-IFNAR1 mAb 48 h or 24 h before infection, respectively. Of note, 17% of animals of the group that received no anti-IFNAR1 mAb developed a mild disease. These results suggest a higher intrinsic pathogenicity of our strain of MAYV in this model compared to CHIKV. Viral loads in animals that received anti-IFNAR1 mAb at any concentration or time point before infection were generally variable, but higher than in animals that received no mAb (Figure 4(e)). All animals that received no anti-IFNAR1 mAbs were viremic but with viral loads not exceeding 10^10^copies/mL.

In a second experiment, we evaluated the effect of 0.1 mg per mouse of anti-IFNAR1 mAb with two viral doses (10^3^ and 10^5^ TCID_50_) (Figure 5(a)). Blood was sampled at two and four dpi. No death or severe disease was observed, and symptom onset occurred between 4 and 7 dpi (Figure 5(b)). All mice receiving the higher viral dose developed mild disease, in contrast to only 33% of those receiving the lower dose. Positive viral loads were observed in most infected animals with a variability which reduced in mice receiving the higher viral dose (Figure 5(c)), and seroconversion was observed in 100% of mice within three weeks (Figure S8(e)).

We further assessed the condition of 0.1 mg anti-IFNAR1/mouse 48 h before infection with 10^5^TCID_50_ of MAYV in mature adult mice (Figure 5(d)). A control group consisting of 7-week-old mice was included. Animals were bled at 3 dpi and monitored daily for 21 days for signs of viral disease. In contrast to our observations with CHIKV, differences were noted between young and adult mice following MAYV infection (Figure 5(e,f)). Adult mice exhibited significantly lower viral loads and a smaller proportion displayed mild clinical symptoms (33% versus 100%), although this difference did not reach statistical significance.

### Low susceptibility of mice to ONNV

ONNV has been reported to be less virulent than CHIKV and MAYV in murine models [12,28], exhibiting lower viral loads compared to those observed with CHIKV and MAYV [29]. We, therefore, infected animals with a high viral dose (10^5^TCID_50_) and tested three anti-IFNAR1 mAb dosing regimens: 0.1 mg or 1 mg administered 48 h before infection, and 1 mg administered 48 h before infection followed by an additional 0.5 mg dose at 1 dpi to ensure continued blocking of IFNAR1. An additional group received 0.1 mg anti-IFNAR1 and was infected with a lower viral dose of 10^4^TCID_50_ (Figure 6(a)). We also had a group of animals receiving no anti-IFNAR1 mAb and the same viral dose. We used the same experimental approach applied to CHIKV and MAYV. Groups of 7-week-old female C57BL/6 mice received anti-IFNAR1 mAb (ip) before infection (ip) with ONNV strain ONNV/UNK/SN/Dakar-234. Blood viral loads were measured at 2 and 4 dpi, and mice were monitored daily for 21 days. On the basis of a clinical assessment grid, survival curves were generated (Figure 6(b)). Even with the high mAb doses, the results confirmed the limited susceptibility of mice to this virus under the tested conditions. Specifically, only one mouse developed symptoms, and low and variable viremia were detected only when anti-IFNAR1 mAb were administrated regardless of the dose (Figure 6(c)). The only notable outcome was that 100% of the mice treated with anti-IFNAR seroconverted, in contrast to only 16% in the group that received the virus alone (Figure S8(g)).

### Viral replication in organs and blood cytokine profiles following CHIKV, MAYV, and ONNV infection

To evaluate our final model, we measured in a last experiment the viral replication in various organs (brain, heart, liver, spleen, small intestine and large intestine) and the serum concentrations of inflammatory cytokines and chemokines (IFN-α, IFN-γ, MCP-1, IL-6, and TNF-α) at 3 and 4 dpi (i.e. peak of viremia). We used the conditions under which we had observed high viral loads with low variability paired with symptomatic disease: 7-week-old female C57BL/6 mice received 0.1 mg anti-IFNAR1 mAb (ip) followed by infection (ip) with 10^5^TCID_50_ of CHIKV, MAYV or ONNV 48 h later. For each virus, we had a group of animals receiving no anti-IFNAR1 mAb. We also had a control group of uninfected animals receiving no anti-IFNAR1 mAb (mock). Groups of 6 mice were euthanized at 3 and 4 dpi (Figure 7(a)).

For CHIKV and MAYV, mice that received the anti-IFNAR1 mAb exhibited increased viral loads in brain, heart, liver and spleen relative to controls with no mAb treatment (Figure 7(b–g)). For CHIKV, significant differences were observed in all tested organs at both 3 and 4 dpi. For MAYV, significant differences could be observed in all tested organs except the small intestine at 3 dpi and in the brain and liver at 4 dpi. Undetectable or very low viral loads were observed in all ONNV-infected mice, with a slight non-significant trend toward higher levels with anti-IFNAR1 mAb-treated animals (Figure 7(f,g)).

Serum concentration of cytokines following infection was measured and compared to the mock group, as well as between groups receiving anti-IFNAR1 mAb or not. For CHIKV, all animals exhibited significant elevation of IFN-α, IFN-γ and MCP-1 concentrations compared to the mock group at both 3 and 4 dpi (Figure 8). Similarly, for MAYV concentrations of these three cytokines were elevated at least on one of the two days, with a significant increase in concentrations observed at 4 dpi for IFN-α and 3 dpi for IFN-γ and MCP-1. With ONNV, a more moderate increase in IFN-α, IFN-γ and MCP-1 levels was observed. The only significant increase in cytokine concentrations after ONNV infection was IFN-α at 3 dpi. For all viruses, almost all concentrations of IL-6 and TNF-α measured were below the limit of detection (Figure S10).

## Discussion

This work proposes a simple approach to refine the pathology of mouse models of arboviruses, more specifically, arthritogenic alphaviruses. Partial immunosuppression through low-dose administration of anti-IFNAR1 mAb was employed to modulate alphavirus-induced disease. This approach, based on a widely available mouse strain, a commercially accessible mAb, and a straightforward protocol, is easily adaptable and could be valuable for studying other viral pathogens.

An interferon response, primarily involving IFN-I, has been shown to enhance survival *in vivo* and effectively limit alphaviral RNA persistence, which is associated with chronic arthralgia [30,31]. The used mAb recognizes an epitope of the IFNAR1 subunit of the receptor recognizing IFN-I. IFN-I activate various immune cells like dendritic cells or macrophages and initiates a signalling cascade in various cell types, leading to the expression of a variety of IFN-I-stimulated genes promoting an antiviral state [32]. *In vivo,* the anti-IFNAR1 mAb MAR1-5A3 induces immunosuppression by blocking IFN-I receptors, rendering mice more susceptible to infection. Various models of viral infection have been described using this mAb [14,15,33,34]. The dose recommended by the manufacturer to fully saturate the receptor is 2.5 mg per mouse as a loading dose, with a maintenance dose of 0.5 mg per week. However, most studies reported that a loading dose of 1 mg is sufficient to obtain immunosuppression. Lower doses have also been described with Zika virus (0.5 mg) [35] and lymphocytic choriomeningitis virus (0.25 mg) [36]. The pharmacokinetics of this mAb are not fully characterized, with the notable feature that its serum half-life is shorter at lower doses (≈1.5 days) compared to higher doses (≈7 days). According to the developers, this may be due to large intracellular pools of IFNAR-1 in certain cells that cycle to the surface and bind the antibody. The accelerated clearance from the bloodstream at lower concentrations could be due to the initial saturation of these intracellular IFNAR1 pools [18].

In this study, we used different sub-saturating doses of an IFNAR1 mAb to explore the effect on disease severity of different alphaviruses. Typically, CHIKV and MAYV do not induce any symptoms in WT mice after ip infection [37]. For both CHIKV and MAYV, we were able to induce mild disease with an antibody dose as low as 0.1 mg. Increasing the dose to 0.3 mg or 1 mg led to more severe disease, with mortality rates of 66% and 83%, respectively, indicating a dose-dependent effect. Additionally, higher viral inocula reduce intra-group variability in terms of blood viral load and clinical signs.

We performed a more detailed characterization of the model using the following parameters: administration of 0.1 mg per mouse of anti-IFNAR1 mAb, followed 48 h later by infection with CHIKV, MAYV, or ONNV at a dose of 10^5^ TCID_50_. For CHIKV and MAYV, this model closely resembles the acute phase of infection observed in humans and non-human primates (NHPs) [38,39]. It induces moderate but measurable clinical illness lasting several days. As in humans and NHP models, viremia is high and viral loads are detected in various organs. Seroconversion is observed in all animals between 2- and 3-week post-infection. ONNV is less pathogenic than CHIKV and MAYV in mice. For example, CHIKV and MAYV quickly induce death in IFNAR-KO mice (A129 mice) [40] while ONNV infection results in mortality rates of 50–100% [12,28]. In this study, mice did not develop any symptoms after infection, even after administration of a high dose of mAb (1 mg) and a secondary follow-up dose (0.5 mg) paired with a high viral dose. The conditions presented here are thus not suitable for modulating ONNV infection due to the low susceptibility of mice to infection. A recent study of Weber *et al.* suggested newer ONNV strains may exhibit higher pathogenicity in mice [41]. Yet interestingly, all mice infected with the highest dose of ONNV and receiving the anti-IFNAR1 mAb seroconverted, whereas only 16% of mice that did not receive the mAb seroconverted, indicating a potential role of the antibody in enhancing viral susceptibility.

During alphavirus infections, a wide range of cytokines and chemokines are upregulated, including IFNs, chemokines, interleukins, colony-stimulating factors, and cytokines of the TNF superfamily [42–45]. In our model, we measured various blood cytokines and chemokines at 3 and 4 dpi. We detected elevated serum concentrations of IFN-α, IFN-γ, and MCP-1 in CHIKV and MAYV-infected mice. IL-6 and TNF-α could not be detected. The observed levels of IFN-α, IFN-γ, and MCP-1 are largely consistent with findings from infected patients [42–45], NHP models [39,46–48], and the arthritis/myositis model [11]. However, reduced MCP-1 levels have also been reported in some CHIKV-infected patients [49]. Concerning IL-6 and TNF-α, however, studies are discordant. Several studies detected elevated levels of one or both cytokines shortly after infection or symptom onset [42,43,45,48–51], while others did not detect significant increases of one or both of these cytokines [46–50]. In our experiments, blood sampling was performed at 3 and 4 dpi, which corresponds to the period just before the onset of clinical symptoms. It is, therefore, possible that IL-6 and TNF-α levels increase only after symptom onset.

While they are mostly known for causing arthralgia and fever, CHIKV and MAYV can cause a wide range of other clinical manifestations. In our final model, we detected high CHIKV and MAYV loads at 3 and 4 dpi in almost all sampled organs (the brain, heart, liver, spleen, large- and small-intestine). This broad tropism is helped by the widespread expression of MXRA8, the receptor for arthritogenic alphaviruses [52]. Studies exploring the pathophysiology of CHIKV patients with fatal outcomes found CHIKV to cause multi-organ failure affecting all tested organs (brain, heart, lung, liver, spleen and kidneys)[49,53]. Though not considered to be a neurotropic virus, various articles describe neurological complications in CHIKV patients [54,55]. A MCP-1 mediated trojan horse mechanism was proposed as an explanation for CHIKV to cross the blood-brain barrier [49]. MAYV is able to infect human astrocytes [56] and cause brain damage in rhesus macaques [47]. Cardiovascular manifestations have been documented in CHIKV-infected patients, most notably myocarditis [57]. In rhesus macaques, MAYV has been shown to affect the heart, leading to tissue damage [47]. Gastrointestinal manifestations are common among CHIKV-infected patients [54], and in rhesus macaques, CHIKV was shown to infect several gastrointestinal tissues and affect the gastrointestinal microbiome [58].

This study has several limitations. Although clear clinical distress is evident in the infected mice, weight loss is either minimal or not statistically significant. Pathology in joint and muscle tissues in animals infected with this model was not studied. Anti-IFNAR1 mAb and virus doses were not normalized by weight, resulting in a slight variation of doses between individuals, especially in older mice. In this study, only female mice were used. As female mice exhibit a more pronounced Type-I IFN response than their male counterparts, future studies applying the anti-IFNAR1 mAb at sub-saturating doses may need to adjust the anti-IFNAR1 dose separately for male and female mice, depending on the specific study objectives. Furthermore, mice of the ages 7 and 11 weeks were infected. The disease outcome of younger mice with more immature immune systems and older mice is likely to vary. The anti-IFNAR mAb doses in mice models with other age groups would need to be adjusted independently.

In conclusion, our findings demonstrate that the disease severity of CHIKV and MAYV can be modulated using varying doses of anti-IFNAR1 mAb. This approach is easily transferable for eventual use in other murine viral infection models. The two main features of this approach are the possibility to modulate the disease severity and the transient nature of the partial immunosuppression induced through the anti-IFNAR1 mAb. Possible applications using this approach include immunization and subsequent challenge studies with different primary disease severities, and the study of the pathogenesis of mice with different gene KOs. Finally, we also propose a new CHIKV and MAYV infection model which imitates the general kinetics of disease progression and pathogenesis in various organs and complements the existing other models for alphavirus-induced arthralgia. This model can be used for testing of antivirals or vaccines as well as for pathogenesis studies in tissues other than the joints.

## Supplementary Material

Supplementary Tables 2025_05_30.xlsx

Supplementary Figures 2025_08_14.docx

## Data Availability

Data are provided within the manuscript, supplementary information files or can be obtained upon request.
