# Supplementary material for: Modulating Chikungunya and Mayaro virus-induced disease severity in mice using low concentrations of anti-IFNAR1 antibodies
Source: Emerg Microbes Infect. 2026 Jan 14;15(1):2611479. doi: 10.1080/22221751.2025.2611479 (PMC12805855; doi:10.1080/22221751.2025.2611479)
Supplement: Supplementary Figures 2025_08_14.docx [file TEMI_A_2611479_SM4369.docx]

Supplementary Materials

## Modulating Chikungunya and Mayaro virus induced disease severity in mice using low concentrations of anti-IFNAR1 antibodies

Konrad Wesselmann^a#^, Léa Luciani^a,b^, Gregory Moureau^a^, Jean-Selim Driouich^a,b^, Ornellie Bernadin^a^, Magali Gilles^a^, Xavier de Lamballerie^a,b,c^, Antoine Nougairède^a,b^

^a^Unité des Virus Émergents (UVE: Aix-Marseille Univ, Università di Corsica, IRD 190, Inserm 1207, IRBA), France.

^b^Assistance Publique-Hôpitaux de Marseille (AP-HM), Service de Virologie Aiguë et Tropicale, Marseille, France.

^c^National Reference Center for Arboviruses, Inserm-IRBA, Marseille, France.

#Address correspondence to Konrad Wesselmann: [konrad.wesselmann@univ-amu.fr](mailto:konrad.wesselmann@univ-amu.fr)


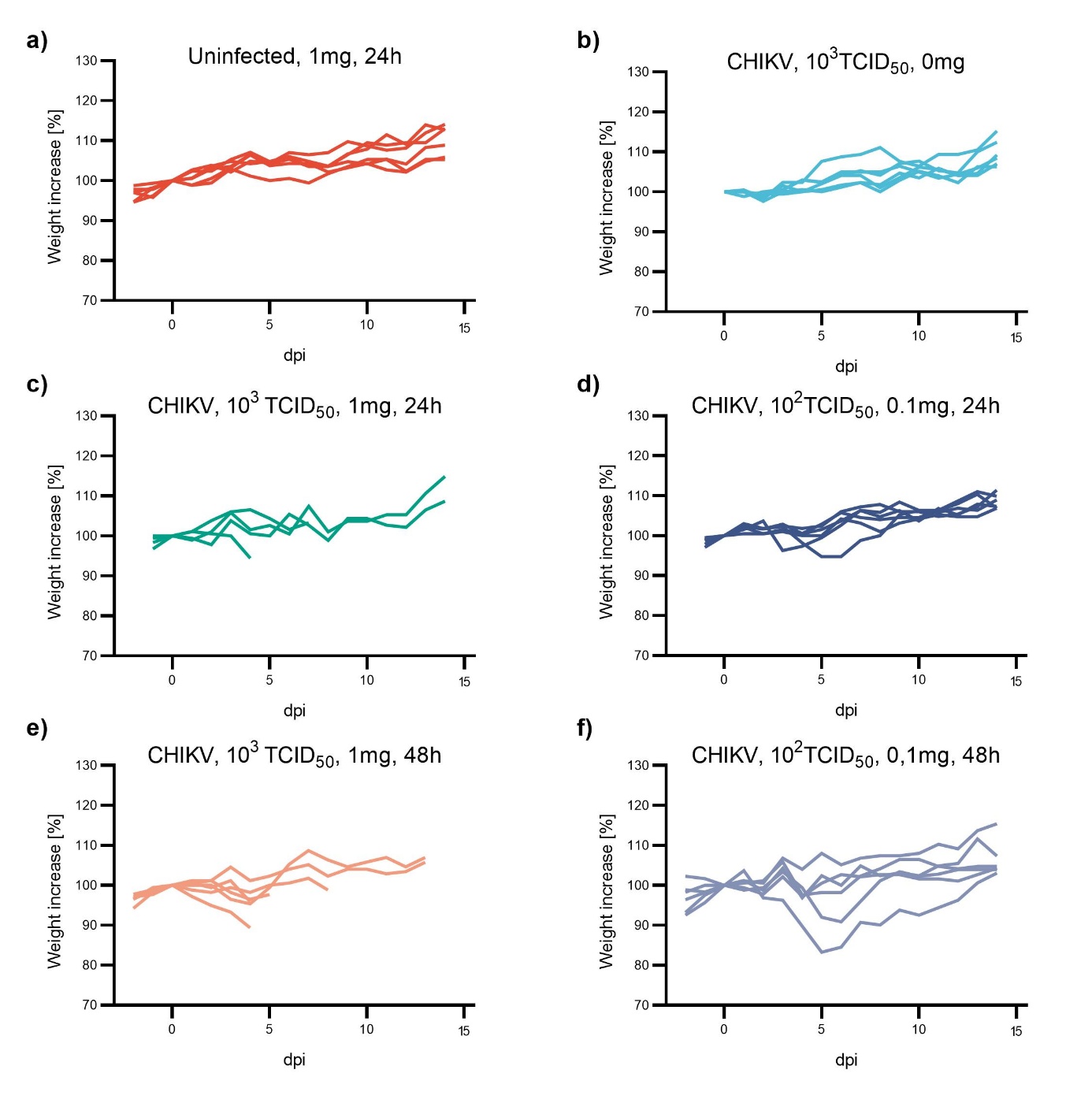


Figure S1: Normalized weights following CHIKV infection with high or low preceding doses of anti-IFNAR1 mAb during experimentation period. Normalized weight was expressed as a percentage of the initial weight (day of infection) calculated as follows: [(weight at day n / weight at day 0)*100]. Data are represented as individual weights of each replicate.


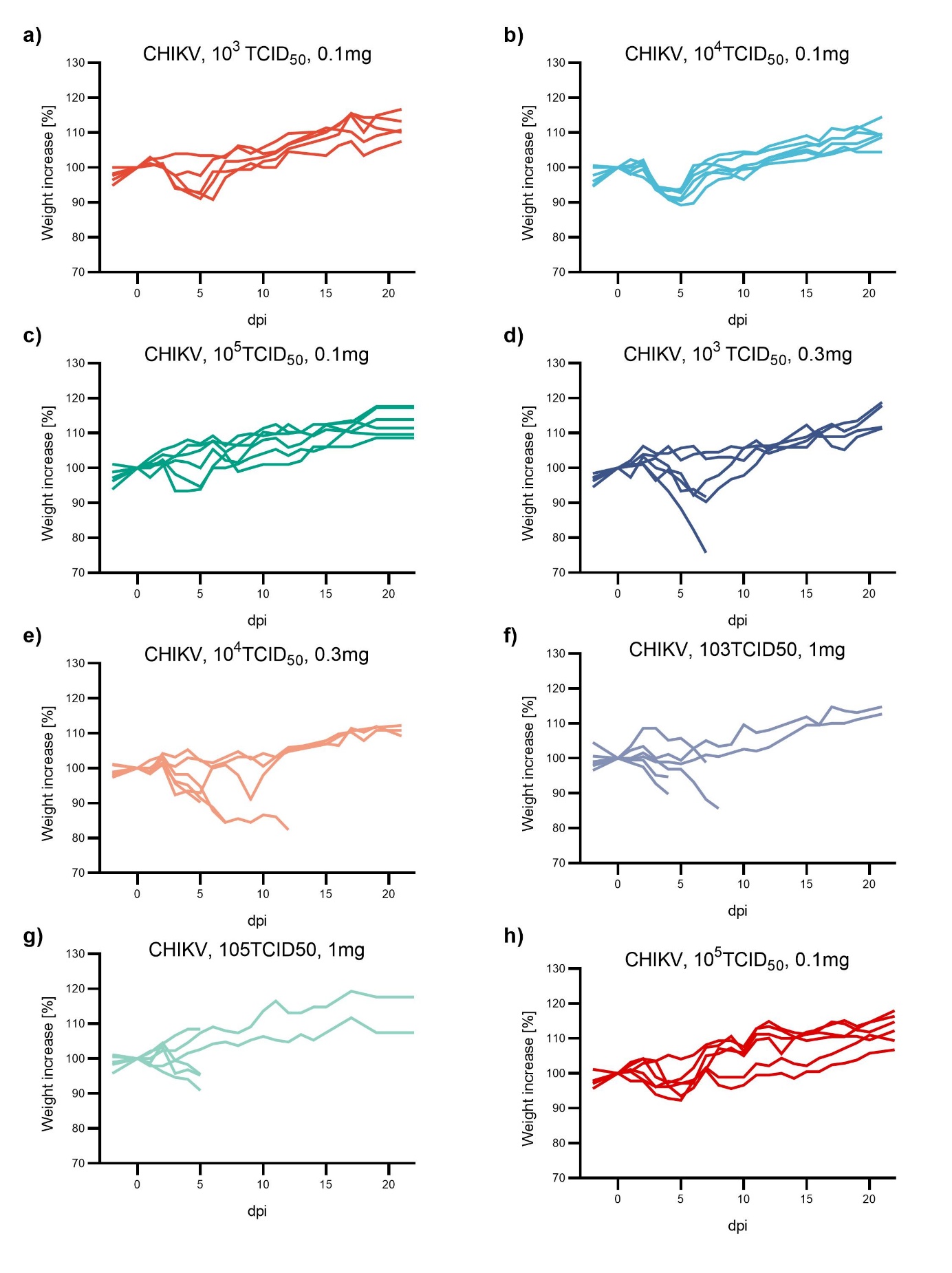


Figure S2: Normalized weights following CHIKV infection with varying infectious doses and varying preceding doses of anti-IFNAR1 mAb during experimentation period. Normalized weight was expressed as a percentage of the initial weight (day of infection) calculated as follows: [(weight at day n / weight at day 0)*100]. Data are represented as individual weights of each replicate.


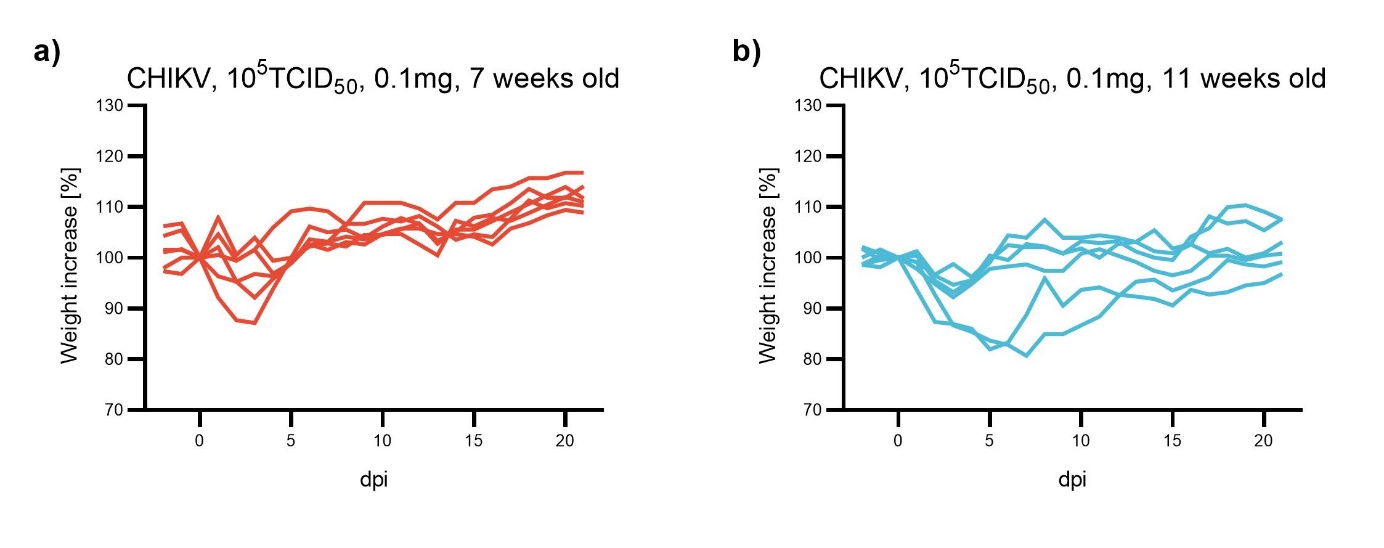


Figure S3: Normalized weights following CHIKV infection in young and mature mice with a preceding dose of 0.1mg anti-IFNAR1 mAb and a high viral titer of 105TCID50 during experimentation period. Normalized weight was expressed as a percentage of the initial weight (day of infection) calculated as follows: [(weight at day n / weight at day 0)*100]. Data are represented as individual weights of each replicate.


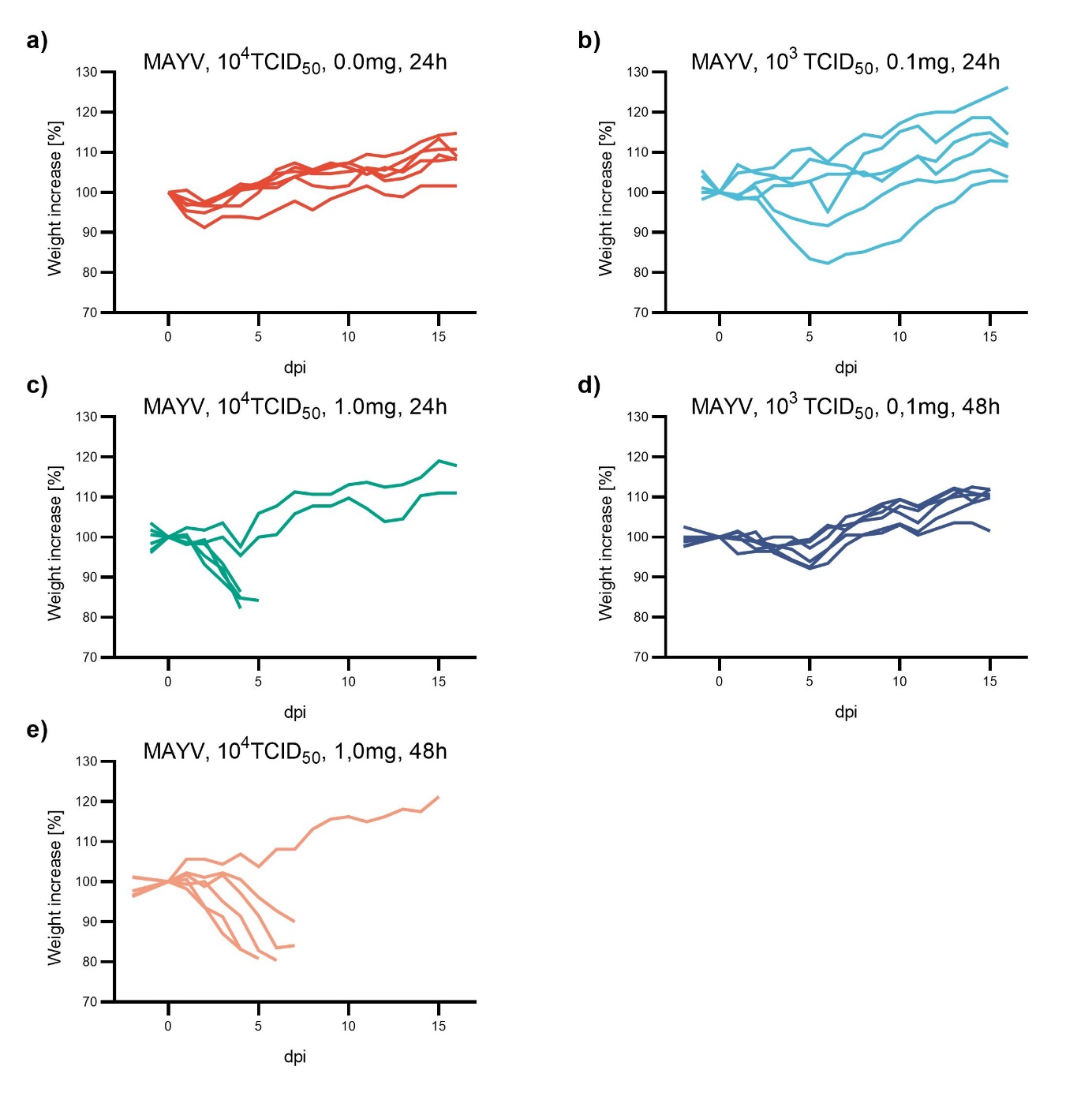


Figure S4: Normalized weights following MAYV infection with very high or very low preceding doses of anti-IFNAR1 mAb during experimentation period. Normalized weight was expressed as a percentage of the initial weight (day of infection) calculated as follows: [(weight at day n / weight at day 0)*100]. Data are represented as individual weights of each replicate.


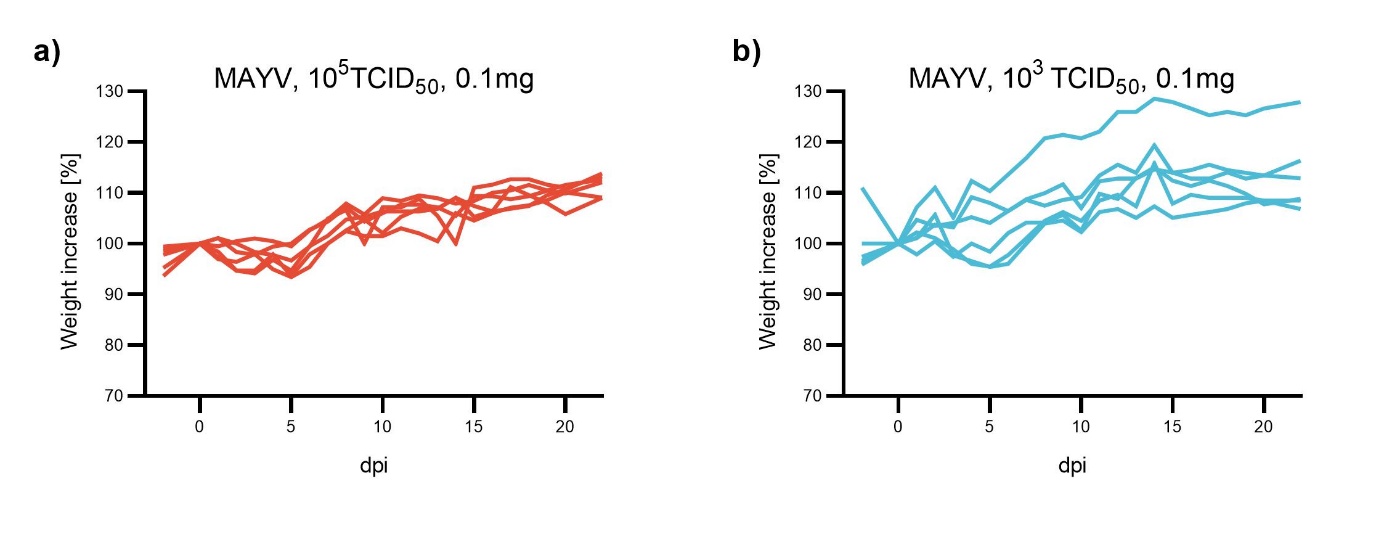


Figure S5: Normalized weights of MAYV infection with 103TCID50 or 105TCID50 and a preceding dose of 0.1mg anti-IFNAR1 mAb during experimentation period. Normalized weight was expressed as a percentage of the initial weight (day of infection) calculated as follows: [(weight at day n / weight at day 0)*100]. Data are represented as individual weights of each replicate.


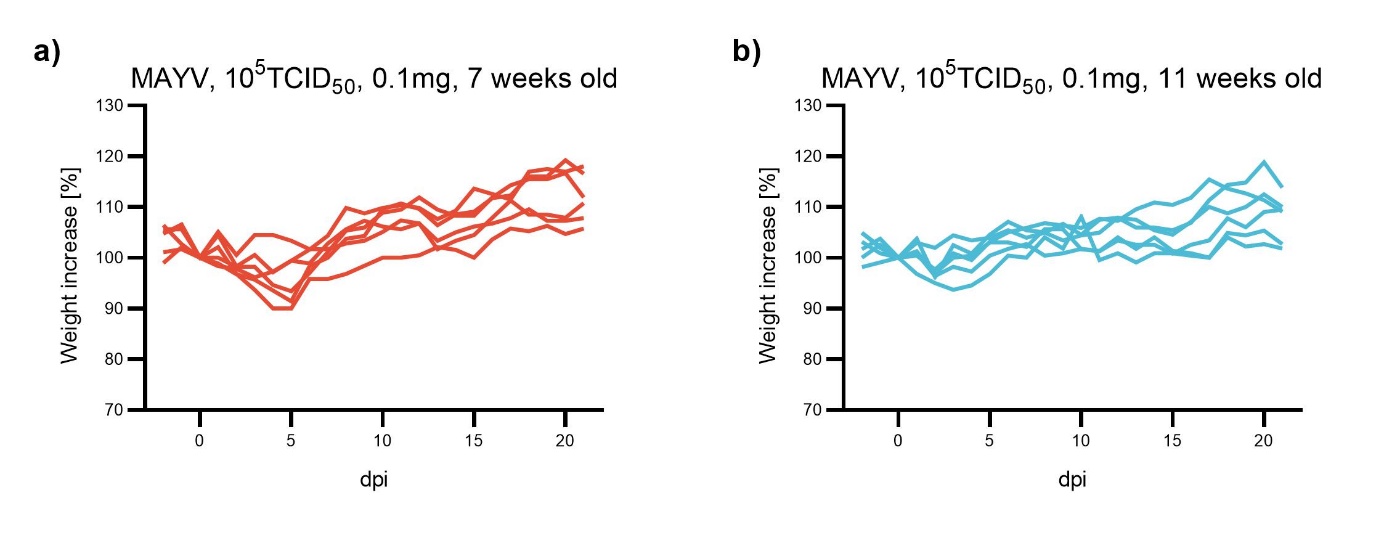


Figure S6: Normalized weights following MAYV infection in young and mature mice with a preceding dose of 0.1mg anti-IFNAR1 mAb and a high viral titer of 10^5^TCID_50_ during experimentation period. Normalized weight was expressed as a percentage of the initial weight (day of infection) calculated as follows: [(weight at day n / weight at day 0)*100]. Data are represented as individual weights of each replicate.


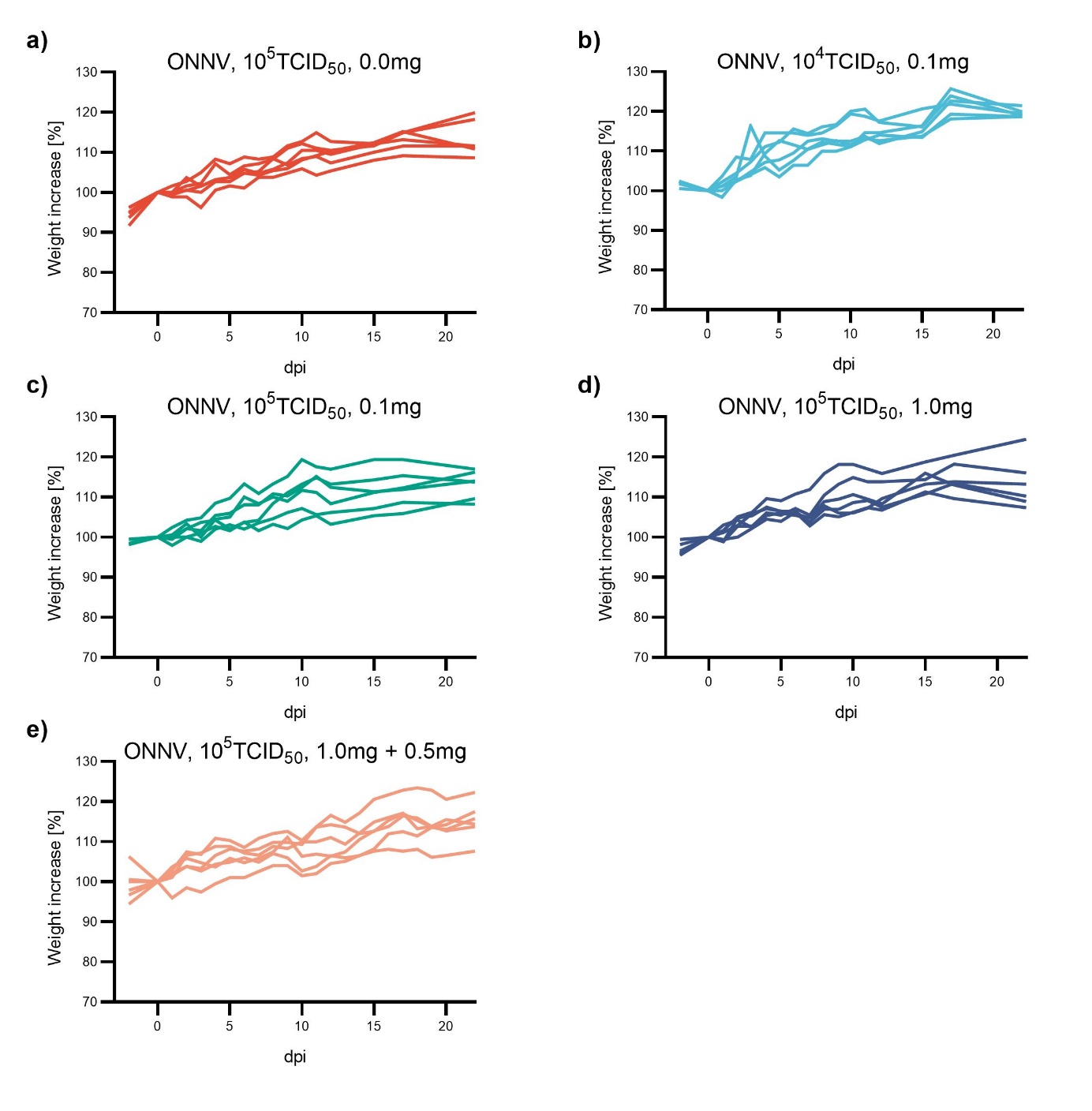


Figure S7: Normalized weights following MAYV infection with 103TCID50 or 105TCID50 and a preceding dose of 0.1mg anti-IFNAR1 mAb during experimentation period. Normalized weight was expressed as a percentage of the initial weight (day of infection) calculated as follows: [(weight at day n / weight at day 0)*100]. Data are represented as individual weights of each replicate.

**
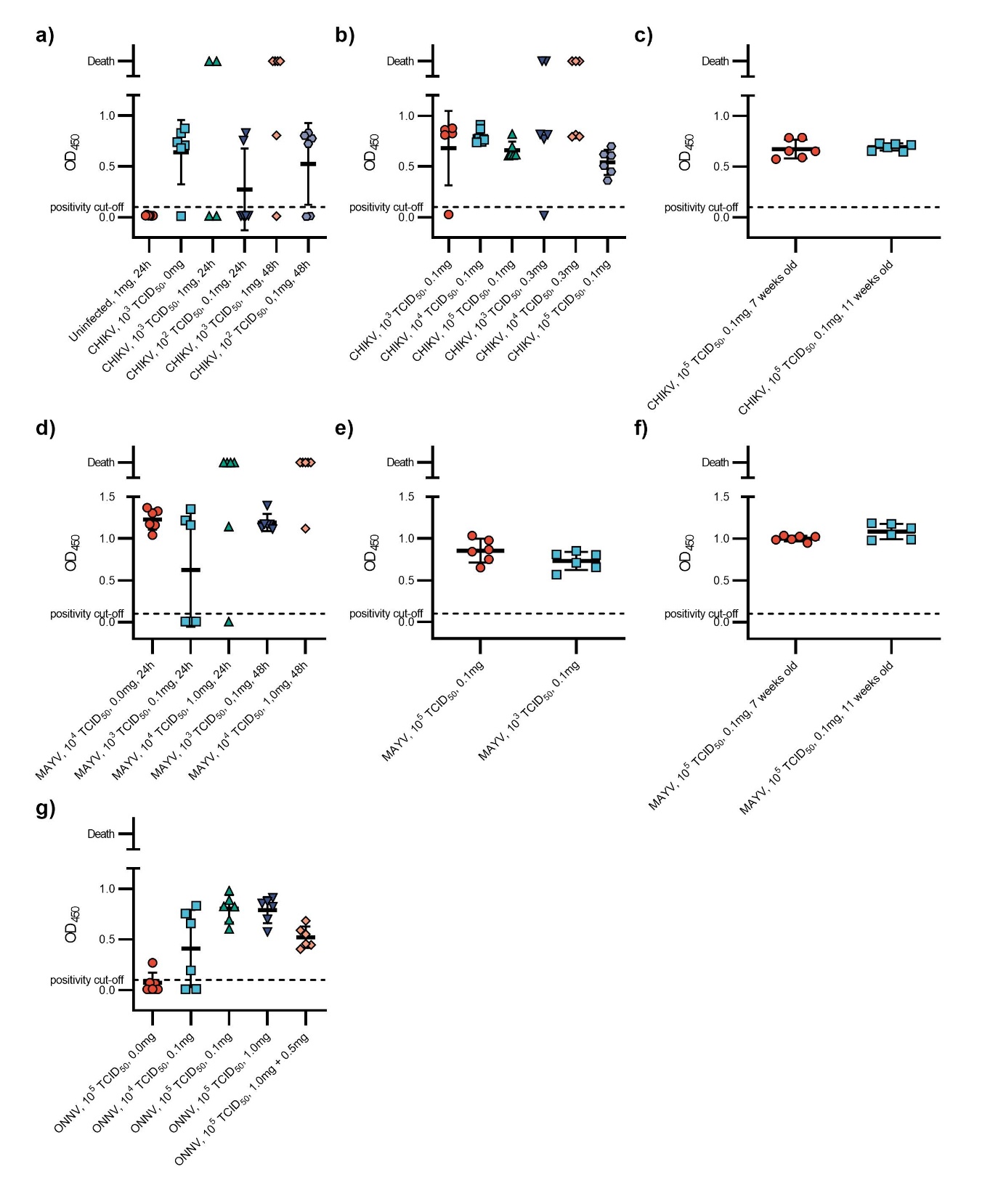
**Figure S8: ELISA results of mice infected with varying doses of CHIKV (a,b and c), MAYV (d, e and f) or ONNV (g) with varying doses of previously injected anti-IFNAR1 mAb (0.1, 0.3, 1 or 1+0.5mg). Virus specific ELISA tests were performed for CHIKV and MAYV. For ONNV a CHIKV-specific ELISA was used. Results are presented as optical density at 450nm.

**
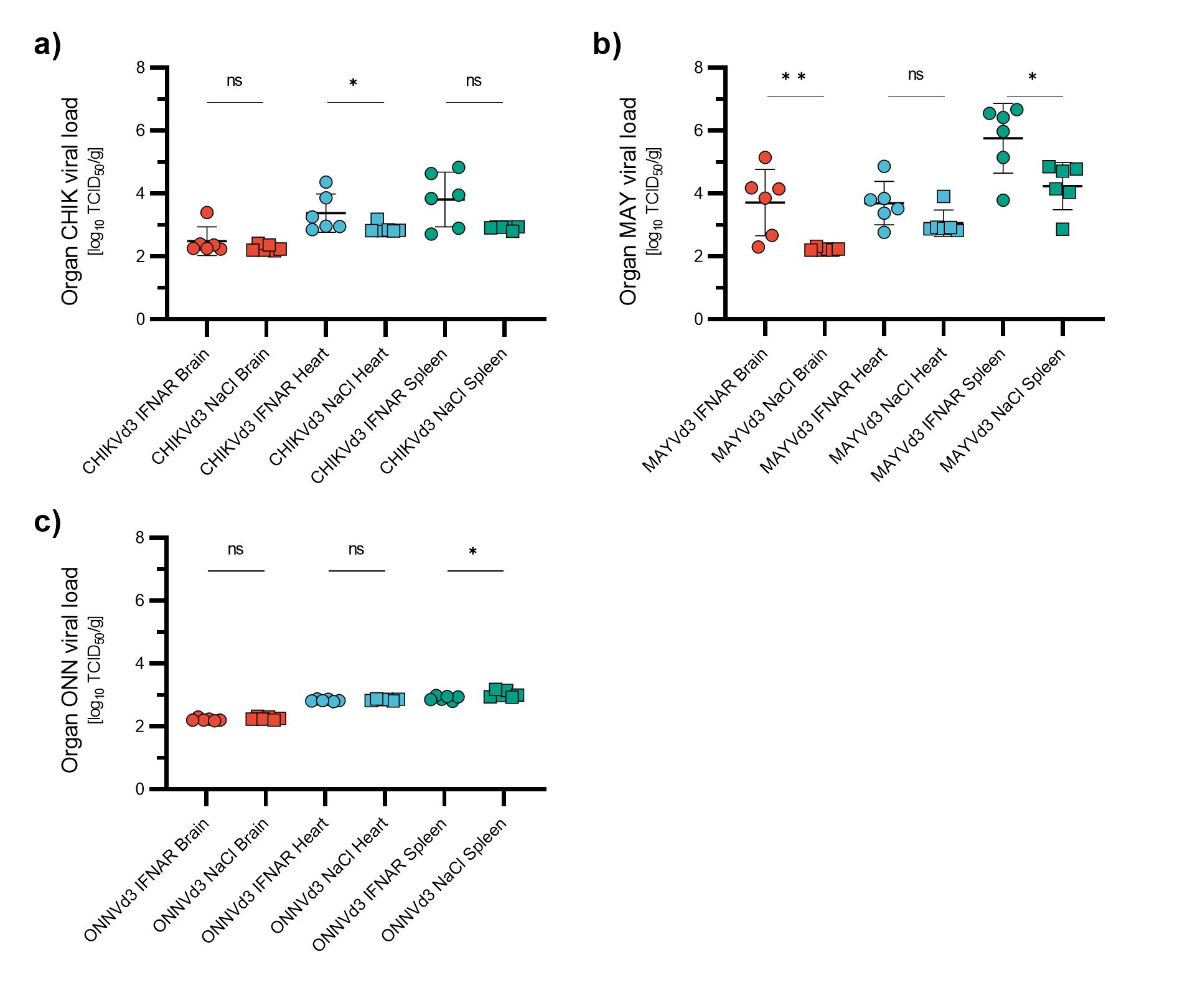
**

Figure S9: Infectious viral loads in various organs after infection with the established model. 7 weeks old C57BL/6J mice were injected with anti-INAR1 mAb (0.1mg) 48h before infection with CHIKV, MAYV or ONNV (10^5^TCID_50_). Mice were euthanized at 3 (a, c and e) or 4 (b, d and f) dpi and TCID_50_ of brain, heart and spleen were determined. At the moment of viral load determination samples had undergone two freeze-unfreeze cycles. Data are represented as mean±SD. Two-sided statistical analysis was performed using Shapiro–Wilk normality test followed by Student t-test, Student t-test with Welch correction or Mann-Whitney test (details in Table S8). *, ** and *** mean p-value ranging between 0.01−0.05, 0.05–0.001 and 0.001–0.0001 respectively. ****: p-value ≤0.0001. ns: not significant.

**
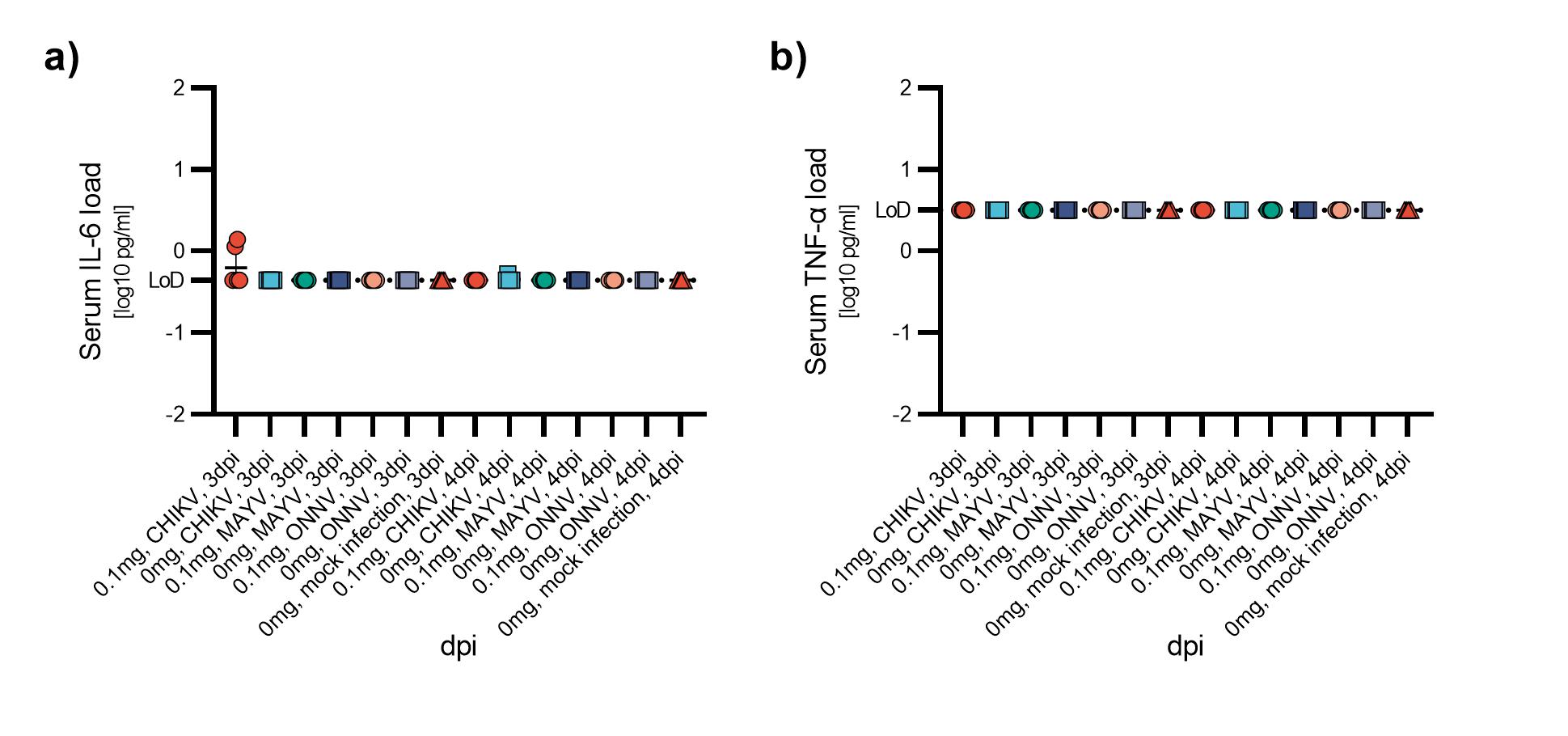
**

Figure S10: Serum IL-6 and TNF-α concentrations after infection with the established model. 7 weeks old C57BL/6J mice were injected with anti-INAR1 mAb (0.1mg) 48h before infection with CHIKV, MAYV or ONNV (10^5^TCID_50_). Mice were euthanized at 3 or 4dpi and serum cytokine concentrations were determined using ProQuantum high-sensitivity immunoassays.
